# RNase L Mediated Protection from Virus Induced Demyelination

**DOI:** 10.1371/journal.ppat.1000602

**Published:** 2009-10-02

**Authors:** Derek D. C. Ireland, Stephen A. Stohlman, David R. Hinton, Parul Kapil, Robert H. Silverman, Roscoe A. Atkinson, Cornelia C. Bergmann

**Affiliations:** 1 Department of Neuroscience, Lerner Research Institute, The Cleveland Clinic, Cleveland, Ohio, United States of America; 2 Department of Pathology, University of Southern California Keck School of Medicine, Los Angeles, California, United States of America; 3 Department of Biological, Geological and Environmental Sciences, Cleveland State University, Cleveland, Ohio, United States of America; 4 Department of Cancer Biology, Lerner Research Institute, The Cleveland Clinic, Cleveland, Ohio, United States of America; The Fox Chase Cancer Center, United States of America

## Abstract

IFN-α/β plays a critical role in limiting viral spread, restricting viral tropism and protecting mice from neurotropic *coronavirus* infection. However, the IFN-α/β dependent mechanisms underlying innate anti-viral functions within the CNS are poorly understood. The role of RNase L in viral encephalomyelitis was explored based on its functions in inhibiting translation, inducing apoptosis, and propagating the IFN-α/β pathway through RNA degradation intermediates. Infection of RNase L deficient (RL^−/−^) mice with a sub-lethal, demyelinating mouse hepatitis virus variant revealed that the majority of mice succumbed to infection by day 12 p.i. However, RNase L deficiency did not affect overall control of infectious virus, or diminish IFN-α/β expression in the CNS. Furthermore, increased morbidity and mortality could not be attributed to altered proinflammatory signals or composition of cells infiltrating the CNS. The unique phenotype of infected RL^−/−^ mice was rather manifested in earlier onset and increased severity of demyelination and axonal damage in brain stem and spinal cord without evidence for enhanced neuronal infection. Increased tissue damage coincided with sustained brain stem infection, foci of microglia infection in grey matter, and increased apoptotic cells. These data demonstrate a novel protective role for RNase L in viral induced CNS encephalomyelitis, which is not reflected in overall viral control or propagation of IFN-α/β mediated signals. Protective function is rather associated with cell type specific and regional restriction of viral replication in grey matter and ameliorated neurodegeneration and demyelination.

## Author Summary

Initial spread of viruses is controlled by type I interferon induced antiviral molecules. Early intervention with viral replication is especially critical in central nervous system infections to reduce loss of non-renewable cells and mitigate immune pathology. One of the best characterized anti-viral mechanisms is mediated by ribonuclease L (RNase L). RNase L exerts activity at multiple levels, including degradation of viral and host RNA, induction of apoptosis, and propagation of the IFN-α/β pathway. Recent studies suggest that RNase L antiviral activity is dependent on the virus, as well as the cell type and tissue infected. This study demonstrates that RNase L protects mice infected with a sub-lethal, demyelinating neurotropic *coronavirus* by ameliorating encephalitis and morbidity, albeit without affecting control of infectious virus or IFN-α/β expression. RNase L specifically protected the brain stem from sustained infection and prevented spread of virus to microglia/macrophages located in spinal cord grey matter. The subtle regional alteration in tropism in the absence of RNase L coincided with increased apoptotic cells and earlier onset as well as increased severity of axonal damage and demyelination. The results demonstrate how subtle regional alterations in viral tropism within the CNS may severely affect the balance between neuroprotection and neurotoxicity mediated by microglia/macrophages.

## Introduction

Type I interferon (IFN-α/β) induced anti-viral responses are critical for the initial control of most virus infections. Although anti-viral responses are initiated in infected cells, they act in an autocrine and paracrine fashion, antagonizing virus replication in host cells and inducing a protective anti-viral state in neighboring cells. The anti-viral state is distinguished by the expression of numerous IFN stimulated genes (ISG), which directly or indirectly interfere with both viral and host RNA transcription and translation [1]. Many of these pathways can also result in the apoptosis of infected cells [2]. Nevertheless, the relative contributions of these various anti-viral effector mechanisms to overall virus control differ with the virus as well as the tissue and cell type infected.

The best characterized anti-viral mechanisms are mediated by activation of double-stranded RNA-dependent protein kinase (PKR) and the 2′-5′-oligoadenylatesynthase (OAS)/ribonuclease L (RNase L) pathways [3]–[5]. Upon recognition of double-stranded RNA, the OAS family of proteins convert ATP into unique, unstable 2′-5′-A oligomers, which are bound by latent RNase L monomers, driving the dimerization and activation of RNase L. Activated RNase L cleaves single-stranded RNA, 3′ of U-A and U-U sites found in both viral and cellular RNA. RNase L endoribonuclease activity is thus not limited to viral RNA, but also degrades host cell RNA, including ribosomal RNA. As OAS genes are stimulated by IFN-α/β, elevated OAS levels enhance the anti-viral state in adjacent, non-infected cells responding to IFN-α/β. Nevertheless, inappropriate activation of RNase L is counterbalanced by the unstable nature of 2′-5′A oligomers and the endogenous RNase L inhibitor, RL1 [6]. A novel aspect of the OAS/RNase L pathway is the cleavage of host RNA releasing free 3′-monophosphates that can activate the cytoplasmic RNA helicases RIG-I and Mda-5 [7]. Recognition by these pattern recognition receptors initiates IFN-β transcription, similar to the activation mediated by recognition of specific viral RNA structures. RNase L thus not only exerts anti-viral effects via RNA degradation and apoptosis, but also has the capacity to amplify and prolong expression of anti-viral genes and other ISGs.

The anti-viral activity of the OAS/RNase L system has been studied in several virus infections *in vitro* and *in vivo* [3],[4]. Protective mechanisms of the OAS/RNase L pathway are generally more evident for RNA relative to DNA virus infections. However, the contribution of RNase L to disease severity and viral control varies significantly depending on the virus and even virus strain studied, presumably reflecting the diverse mediators and targets of these anti-viral mediators. For example, encephalomyocarditis virus (EMCV) replication was only modestly increased in RNase L deficient (RL^−/−^) mouse embryonic fibroblasts, consistent with enhanced susceptibility to infection *in vivo*. Although RNase L prolonged survival, mortality rates of infected RL^−/−^ and control mice were similar. Furthermore, IFN-β treatment increased the survival time of infected wild-type (wt) and to a lesser extent RL^−/−^ mice, indicating that alternate IFN-dependent anti-viral mechanisms act in the absence of RNase L [8]. In contrast, the requirement for RNase L activity to protect from another *picornavirus*, Coxsackievirus B4, was evident by significantly increased mortality rates of infected RL^−/−^ compared to wt mice. However, increased mortality was not the result of uncontrolled virus replication, as virus titers were only slightly elevated within pancreatic islet cells *in vivo*. By contrast, pancreatic islet cells derived from RL^−/−^ mice, treated with IFN-α and infected *in vitro* exhibited increased virus replication and loss of cellular integrity, compared to IFN-α treated RNase L sufficient cells [9]. These results revealed distinct differences in direct anti-viral functions of RNase L *in vitro* and *in vivo*. Cell type specific effects of anti-viral RNase L activity are also clearly evident from studies with West Nile virus (WNV). While mouse embryonic fibroblasts display RNase L dependent anti-viral activity [10], IFN-β treatment revealed no affect of RNase L in reducing WNV replication in either cortical or peripheral motor neurons [11]. Nevertheless, a modest effect was observed in bone marrow derived macrophages, consistent with increased viral burden in CD11b^+^ splenocytes derived from RL^−/−^ compared to wt mice [11]. Furthermore, increased mortality of RL^−/−^ mice in response to peripheral WNV infection correlated with increased replication in lymphoid tissue, but not enhanced viral dissemination into the CNS. A minor role for RNase L in control of WNV in the CNS was supported by similar viral titers in brains as well as kinetics and rates of mortality following intracranial infection of RL^−/−^ and wt mice. Although viral replication was only transiently increased in spinal cords of intracranially infected RL^−/−^ mice, the most prominent effect was increased spread to the spleen and liver [11]. The more critical role of RNase L in limiting WNV spread in peripheral tissues rather than the CNS highlights the complexities of OAS/RNase L mediated anti-viral mechanisms *in vivo*.

IFN-α/β mediated innate responses are also essential to control virus replication and survival following *coronavirus* infections [12]–[15]. Infections *in vitro* and *in vivo* induce upregulation of IFN-α/β and anti-viral mediators, including PKR and OAS [12],[16],[17]; however, the participation of these pathways in the anti-viral response to CNS infection has not been elucidated. *In vitro*, the dual liver and neurotropic MHV-A59 strain does not elicit degradation of 18S and 28S ribosomal RNA, suggesting the absence of RNase L activation in HELA cells [18]. However, administration of an RNase L agonist inhibited replication of the hepatotropic MHV-3 strain in peritoneal macrophages *in vitro* and in liver *in vivo* [19]. Nevertheless, reduced viral replication via RNase L function *in vivo* was insufficient to prevent liver necrosis and death. The nonlethal gliotropic JHM strain of mouse hepatitis virus (MHV-JHM) replicates exclusively in the CNS following intracranial infection and is controlled by both innate and adaptive responses [20],[21]. CNS infection results in a non-lethal encephalitis with immune-mediated demyelination resulting in hind limb paralysis [21]. Based on expanded tropism to hippocampal neurons and widespread dissemination within the CNS of mice deficient in IFN-α/β signalling (IFNAR^−/−^) [12], the gliotropic MHV-JHM variant was used to probe an anti-viral role of RNase L within the CNS. While the vast majority of wt mice survived, RL^−/−^ mice succumbed to infection, albeit delayed compared to IFNAR^−/−^ mice. Although enhanced morbidity suggested a prominent role of RNase L in anti-viral protection, the absence of RNase L neither diminished overall virus control in the CNS, nor enhanced neuronal infection. Furthermore, RL^−/−^ mice did not reveal any impairment in IFN-α/β induction, alterations in proinflammatory cytokines or inflammation compared to wt mice. These data rather reveal a novel role of RNase L in specifically counteracting focal microglia/macrophage infection, thus potentially sustaining microglia mediated neuroprotective effects and ameliorating demyelination.

## Results

### RNase L deficiency increases morbidity without affecting virus control

**Figure 1:**
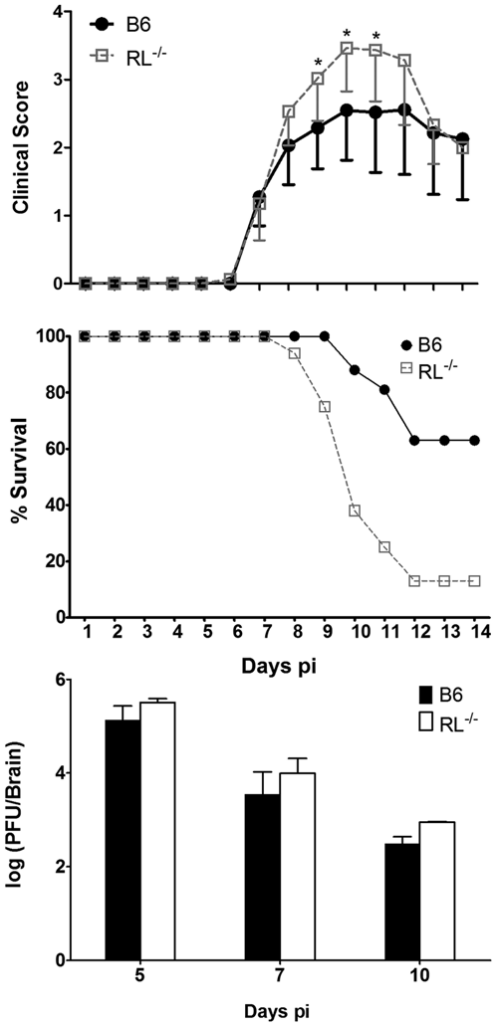
RNase L ameliorates clinical symptoms and is required for recovery from MHV-JHM. Top Panel: Morbidity of wt and RL^−/−^ mice infected with gliotropic MHV-JHM. Data are the average of two independent experiments. Middle Panel: Survival rates of wt and RL^−/−^ (n = 16/group) following i.c. infection with MHV-JHM. Data are representative of two independent experiments. Bottom Panel: Virus replication in the brains of infected mice is not significantly altered by RNase L deficiency (n = 6/group).

Mice deficient in IFN-α/β signalling succumb to an otherwise sub-lethal gliotropic MHV-JHM within 8 days of infection despite functional CD8 T cell responses [12]. Uncontrolled viral replication in the absence of IFN-α/β implicated a crucial role of anti-viral innate mechanisms in stemming viral spread within the CNS. To elucidate the role of RNase L in CNS pathogenesis, gliotropic MHV-JHM infection was examined in RL^−/−^ mice [8]. Increased susceptibility of RL^−/−^ mice was evident by more severe clinical symptoms of acute encephalitis at 9–11 days post infection (p.i.) (Fig. 1, top panel). With the exception of increased severity, onset and progression of clinical symptoms in infected RL^−/−^ were similar to those exhibited by infected wt mice. These included initial symptoms of acute viral encephalitis, including: lethargy, dehydration and weight loss, which progressed to diminished hind limb function. Furthermore, mortality rates were significantly higher in RL^−/−^ mice, with over 90% succumbing to infection by 12 days p.i. (Fig. 1, middle panel), approximately 3–4 days delayed relative to IFNAR^−/−^ mice [12]. Assessment of direct anti-viral functions of RNase L [9],[11] revealed no significant changes of infectious virus in brains of RL^−/−^, compared to wt mice. Virus titers were increased less than 0.5 log_10_ in RL^−/−^ mice (Fig. 1, bottom panel). Very modest anti-viral RNase L activity in the CNS was confirmed by analysis of viral RNA from CNS tissue. Levels of viral mRNA encoding the viral nucleocapsid protein were increased less than 2.4-fold in spinal cords of RL^−/−^ compared to wt mice at any time point (Table 1). As liver may constitute an extraneural infection site in immunocompromised mice, viral replication in this tissue was also assessed by real-time PCR. The abundant viral mRNA encoding the nucleocapsid protein was detected in liver at very low levels compared to the CNS in both groups, albeit only at early time points (Table 1). Although viral RNA was elevated in livers of RL^−/−^ mice at day 7 p.i., clearance by day 10 p.i. suggested hepatitis did not contribute to mortality. Necrotic foci were not evident in either mouse group by gross examination. A minor role of RNase L in viral control *in vivo* was reminiscent of the previously described *picornavirus* models [8],[9],[11].

**Table 1:** Levels of viral nucleocapsid protein encoding mRNA in CNS and liver.

Day p.i.	Spinal cord		Liver	
	wt	RL−/−	wt	RL−/−
4			9.0±2	3.0±2
5	794^a^±44^b^	1085±215		
6			0.5±.03	6.0±.05
7	1769±125	3148±363		
10	613±184	353±124	BD^c^	BD

a relative to GAPDH.

b SD from 3 individual mice.

c below detection.

### RNase L deficiency does not impair type I IFN responses in the CNS

**Figure 2:**
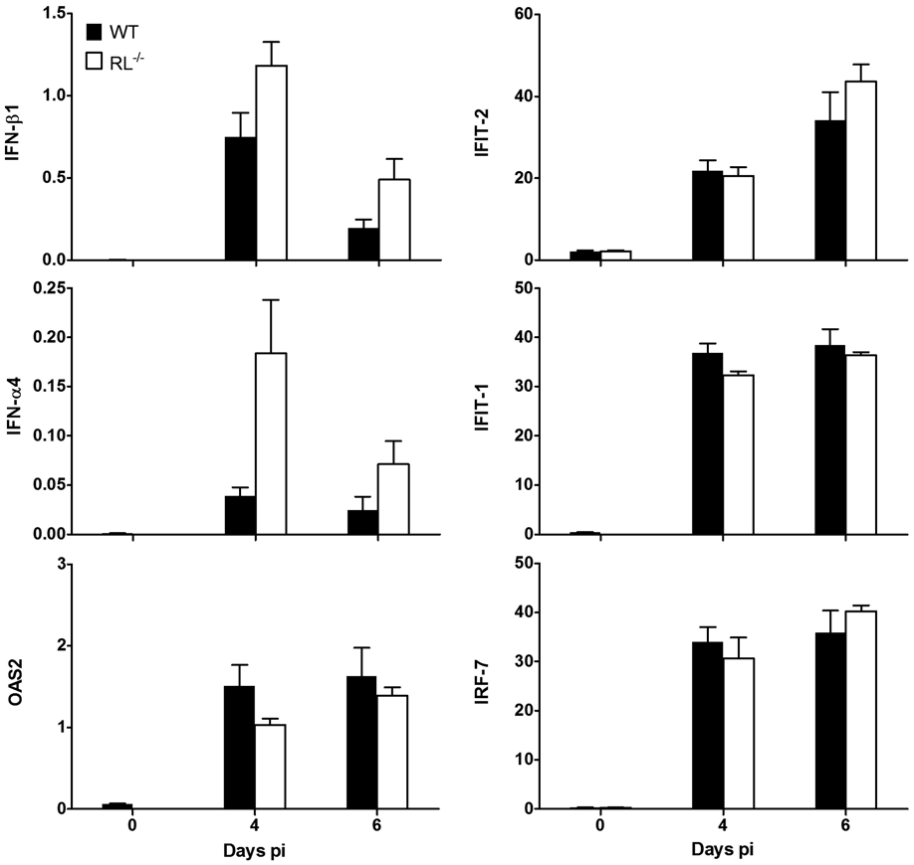
RNase L does not alter IFN responses to MHV-JHM infection of the CNS. Expression of IFN-α4, IFN-β1, OAS2, IFIT-1, IFIT-2 and IRF-7 mRNA relative to the housekeeping gene GAPDH in brains of MHV-JHM infected wt and RL^−/−^ mice. Data are the average of individual mouse brains from two independent experiments (n = 6).

In addition to RNA degradation, RNase L also induces a multitude of genes in the IFN-α/β pathways during infections with Sendai virus and EMCV [7]. Thus, both self and virus small RNA products released by RNase L activate cytoplasmic RNA helicases RIG-I and Mda-5 to optimize and perpetuate anti-viral responses. However, IFN-α and IFN-β expression levels were slightly increased, rather than decreased in brains of MHV-JHM infected RL^−/−^ mice relative to wt mice (Fig. 2). Furthermore, OAS-2, IFIT-1, IFIT-2, and IRF-7, representing prominently activated ISGs, were induced to similar, or modestly increased levels in the CNS of infected RL^−/−^ mice. Slightly enhanced IFN-α/β and ISG expression was also observed in spinal cords from infected RL^−/−^ relative to wt mice (data not shown). Consistent with similar virus replication and control in the CNS, neither IFN-α/β nor the expression of downstream ISGs were impaired at the tissue level. These data suggest that a positive RNase L-dependent feedback loop in propagating IFN-α/β signalling is not activated during MHV-JHM infection in the CNS.

### RNase L does not alter CNS inflammatory responses

**Figure 3:**
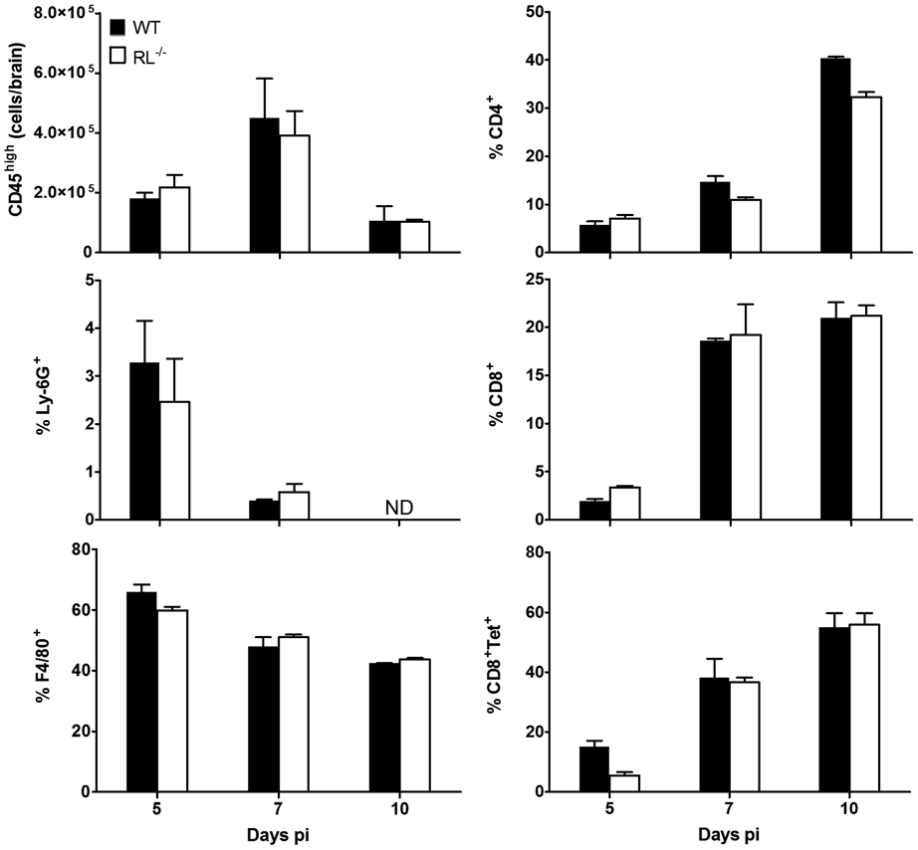
RNase L does not alter CNS inflammation. Numbers and composition of CD45^hi^ inflammatory cells derived from brains of infected wt and RL^−/−^ mice analyzed by flow cytometry at the indicated times p.i. Numbers of bone marrow derived CD45^hi^ cells/brain are shown in the top left panel. Composition of cell infiltrates is indicated by relative percentages of Ly-6G^+^ neutrophils (ND = not detected), F4/80^+^ macrophages, CD4 T cells, and CD8 T cells. Percentages of virus-specific CD8 T cells within the CD8 population is assessed by D^b^/S510 tetramer staining (CD8+Tet+). Data are the mean of two independent experiments±SEM.

An unanticipated feature of RL^−/−^ mice is delayed tissue rejection in transplant studies [22], suggesting a role of RNase L in modifying antigen presentation, lymphocyte trafficking or function. To assure that altered pathogenesis was not due to differential inflammatory responses, the CNS was examined for extent, composition and localization of infiltrating cells. Characterization of cells recruited to the CNS using flow cytometry revealed similar total numbers of infiltrating cells and no alterations in their composition (Fig. 3). Unlike IFNAR^−/−^ mice [12], neutrophil infiltration was not affected in RL^−/−^ mice and macrophages comprised the most prominent population early p.i. in both strains. CD4^+^ and CD8^+^ T cells peaked to nearly identical numbers at day 7 p.i. in both groups. Furthermore, CNS infiltrating CD8^+^ T cells in both groups were comprised of 50% and 60% virus specific cells at day 7 and 10 pi, respectively, as monitored by D^b^/S510 class I tetramer staining [23]. Similarly, no significant differences in the extent of inflammatory cells were observed by histochemical analysis (data not shown). These data confirm that priming and trafficking of virus specific T cells was unaffected by the loss of RNase L activity, consistent with effective clearance of infectious virus.

### RNase L protects the brain stem from sustained MHV-JHM infection and apoptosis

**Figure 4:**
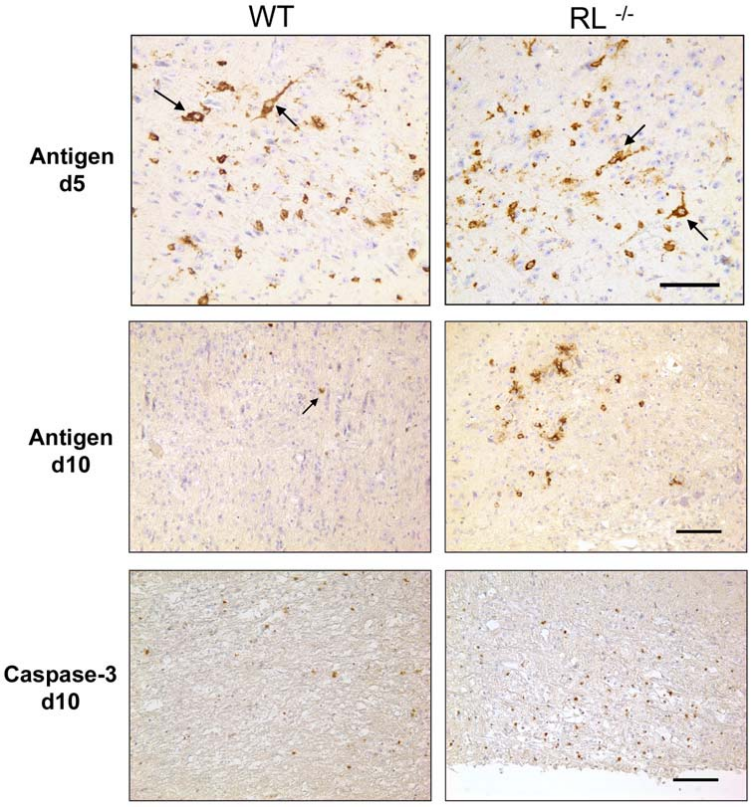
RNase L does not alter the extent of neuronal infection but delays viral clearance from brain stem. Viral nucleocapsid antigen detected by immunoperoxidase staining using mAb J.3.3 in brains from infected wt and RL^−/−^ mice at days 5 and 10 p.i. (top panels: brown chromogen; hematoxylin counterstain). Note neuronal infection (arrows) and similar foci of viral antigen in glial cells in brain stem at day 5 p.i. By day 10 p.i. infection is controlled in wt mice, but foci of infected non neuronal cells with glia morphology are sustained in RL^−/−^ mice. Scale bar = 100 µm. Immunohistochemical staining for activated caspase-3 (bottom panels) at day 10 p.i. indicates increased numbers of cells undergoing apoptosis in brain stem of RL^−/−^ relative to wt mice. Scale bar = 100 µm.

RNase L did not overtly affect overall CNS viral replication; however, it may act in a tissue or cell type specific manner [11]. MHV-JHM initially infects ependymal cells and spreads to astrocytes, microglia/macrophages and predominantly oligodendrocytes, but rarely infects neurons [24]. However, MHV-JHM induced mortality in the absence of IFN-α/β signalling is associated with a dramatically expanded virus tropism to hippocampal neurons [12]. To assess the possibility that RNase L exerts cell type dependent anti-viral effects not apparent from whole tissue homogenates, the distribution of virus infected cells was analysed by immunohistochemistry. Sequential analysis demonstrated a limited infection of neurons within the brain with a predominance in the brain stem at day 5 p.i. in both RL^−/−^ and wt mice (Fig. 4). Glia tropism prevailed in both groups and overall distribution of virus infected cells was similar with occasional neurons still infected at day 7 p.i. (data not shown). By day 10 p.i. virus infected cells declined in cortex and brain stem of both wt and RL^−/−^ mice. However, in contrast to wt mice which had cleared virus from brain stem, virus infected cells were sustained in the brain stem of RL^−/−^ mice and were predominantly microglial in appearance (Fig. 4). The overall extent and distribution of viral infected cells was thus similar during peak virus replication in brains of RL^−/−^ and wt mice, with the exception of sustained infection in brain stem in the absence of RNase L. Preliminary experiments indicate that these cells are microglia or macrophage/monocytes (see below). These data contrast with the infection of IFNAR^−/−^ mice [12] which show predominant neuronal infection. Therefore, preferential infection of neurons could not account for the increased mortality in RL^−/−^ mice. The data also indicated that protective RNase L functions are not overtly reflected in early anti-viral activity but are manifested in region specific protection.

RNase L activation leads to apoptosis and elimination of infected cells, a process requiring activated caspase 3 [3],[25]. Furthermore, IFNα/β mediated activation of RNase L also induces apoptosis in non-infected cells [8]. During MHV-JHM infection of wt mice the majority of apoptotic cells are lymphocytes [26], which are not targets of infection. Although oligodendrocyte apoptosis has been linked to the demyelinating process, the demonstration of apoptotic oligodendrocytes during MHV-JHM infection has been elusive [27],[28]. The retention of virus infected cells in brain stem may result in enhanced local T cell activation and apoptosis of either T cells, infected cells, or both. Therefore, the frequency of apoptotic cells was examined at day 10 p.i. In contrast to the brain stems of wt mice which contained few apoptotic cells, apoptotic cells were prominent in brain stems of RL^−/−^ mice (Fig. 4) coincident with retention of virus infected cells. Although increased focal virus infection associated with substantially increased apoptotic cells presumably dysregulates neuronal function and potentiates tissue damage, there was no evidence that apoptotic cells are preferentially located adjacent to neurons.

### RNase L ameliorates demyelination and axonal damage

**Figure 5:**
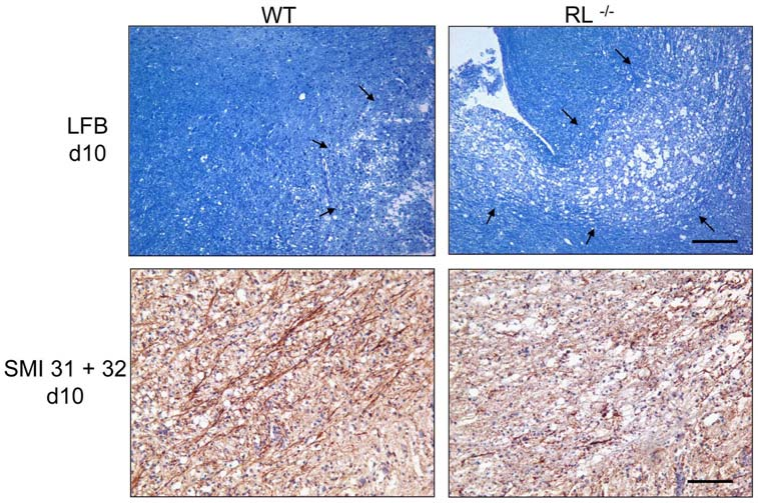
Increased demyelination and axonal damage in brain stem. Brain stems of infected wt and RL^−/−^ mice at day 10 p.i. stained with Luxol Fast Blue (top panels). Scale bar = 100 µm. Immunohistochemical staining for axonal integrity in brain stem at day 10 p.i. (bottom panels) Scale bar = 100 µm. Note more abundant loss of myelin and axonal degeneration in infected RL^−/−^ relative to wt mice.

Sustained brain stem infection and apoptosis in RL^−/−^ mice coincided with increased morbidity and mortality, despite similar overall control of viral replication. The morbidity in MHV-JHM infected RL^−/−^ mice also coincided temporally with the onset of acute primary demyelination in wt mice. RL^−/−^ mice were thus examined for enhanced tissue pathology and demyelination. Brain stems of infected wt mice revealed minimal demyelination by day 10 p.i. (Fig. 5). By contrast, demyelination was more severe in brain stems of RL^−/−^ mice, correlating with increased numbers of virus infected as well as apoptotic cells. Demyelination is associated with a variable degree of axonal damage [29],[30]. Primary immune mediated demyelination in the spinal cord during MHV-JHM infection coincides with early axonal degeneration [31],[32]. While axons were largely intact in brain stems of wt mice, axonal integrity within demyelinated lesions was severely compromised in RL^−/−^ mice as indicated by a striking decrease in neurofilament and an increase in dystrophic axons within lesions (Fig. 5).

**Figure 6:**
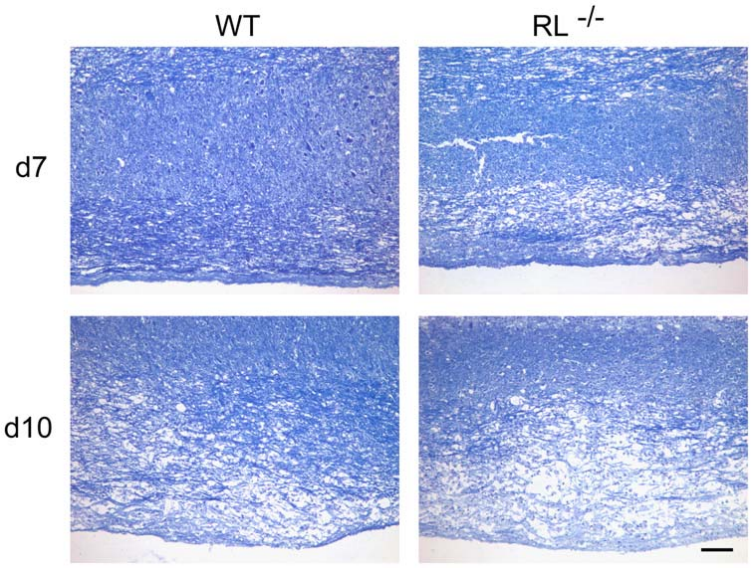
RNase L mitigates spinal cord demyelination. Spinal cords of infected wt and RL^−/−^ mice at day 7 (top) and day 10 (bottom) p.i. stained with Luxol Fast Blue. Scale bar = 100 µm.

In spinal cords of wt mice, demyelination was also minimal at day 7 p.i. and increased by day 10 p.i. (Fig. 6), when control of virus replication is clearly evident (Table 1). By contrast, large focal areas of severe demyelination were already apparent in RL^−/−^ mice at day 7 p.i., resembling those in wt mice at day 10 p.i. In addition to the accelerated kinetics of demyelination, the severity of myelin loss was more pronounced in the absence of RNase L at both days 7 and 10 p.i. Consistent with the more rapid loss of myelin in infected RL^−/−^ mice, quantification of demyelinated areas at day 7 p.i. revealed 1.2±0.8% demyelination in spinal cords of wt mice and 3.0±1.7% in RL^−/−^ mice. Increased demyelination in both brain stem and spinal cord are thus a hallmark of infected RL^−/−^ mice, irrespective of overall viral control.

**Figure 7:**
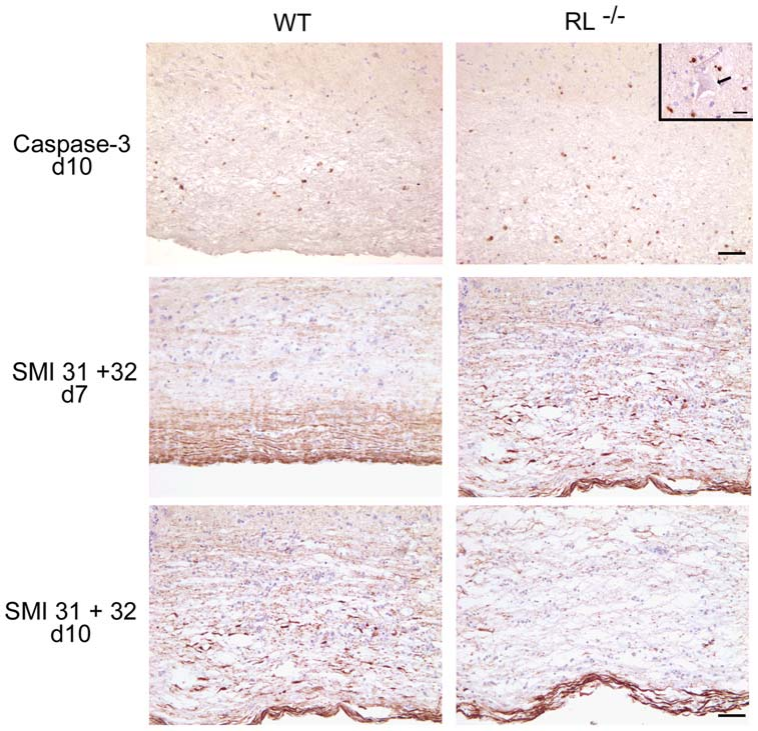
RNase L regulates apoptosis and ameliorates axonal degeneration. Immunohistochemical staining for activated caspase-3 (top panels) at day 10 p.i. indicates apoptosis in grey matter (above line) of spinal cords from RL^−/−^ mice, but not wt mice. Scale bar = 50 µm. Inset shows a large neuron (arrow) surrounded by five apoptotic cells. Scale bar = 20 µm. Immunohistochemical staining for axonal integrity at day 7 (middle panels) and day 10 (bottom panels) p.i. in spinal cord white matter. Note earlier and more abundant loss of axonal integrity in spinal cords from infected RL^−/−^ relative to wt mice. Scale bar = 100 µm.

Apoptotic cell numbers and axonal damage in the spinal cords of RL^−/−^ mice were also evaluated to determine an association with increased demyelination. In wt mice, apoptotic cells were undetectable in spinal cord at day 7 p.i. (data not shown), but were prominent in white matter by day 10 p.i. (Fig. 7). By contrast, spinal cords from RL^−/−^ mice already exhibited small areas of apoptotic cells at 7 days p.i., albeit only in white matter. However, by day 10 p.i. numerous apoptotic cells were evident in both white matter and grey matter of spinal cords from infected RL^−/−^ mice. Furthermore, in contrast to brain stems, apoptotic cells were often detected in close proximity to neurons (inset Fig. 7); however, there was no evidence for neuronal apoptosis. These data suggest that the absence, rather than presence, of RNase L contributes to enhanced apoptosis in white matter, and subsequently also in grey matter.

Analysis of axonal integrity revealed that all axons were intact in spinal cords of wt mice consistent with minimal demyelination at day 7 p.i. (Fig. 7). By contrast, a striking decrease in neurofilament and an increase in dystrophic axons within demyelinated lesions demonstrated axonal integrity was already compromised in white matter of RL^−/−^ mice by day 7 p.i. At day 10 p.i. the amount of axonal degeneration in wt mice resembled that of RL^−/−^ mice at day 7 p.i., consistent with the severity of demyelination. However, by day 10 p.i. spinal cords of RL^−/−^ mice showed marked axonal loss located in areas of myelin loss (Fig. 7), similar to results in brain stem. The increased pathological changes in both brain stem and spinal cord thus correlated with the high mortality rates of RL^−/−^ mice starting at day 9 p.i.

### RNase L prevents viral spread to grey matter microglia/macrophages in spinal cords

**Figure 8:**
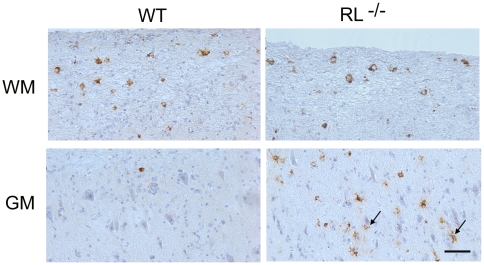
RNase L prevents spread of MHV-JHM to spinal cord grey matter. Viral antigen in spinal cords from infected wt and RL^−/−^ mice at day 7 p.i. Note spread of virus into the grey matter (GM) (arrows, bottom panel) is only observed in RL^−/−^ mice, whereas viral antigen is restricted to white matter (WM) in wt mice. Also note the absence of infected neurons. Scale bar = 50 µm.

**Figure 9:**
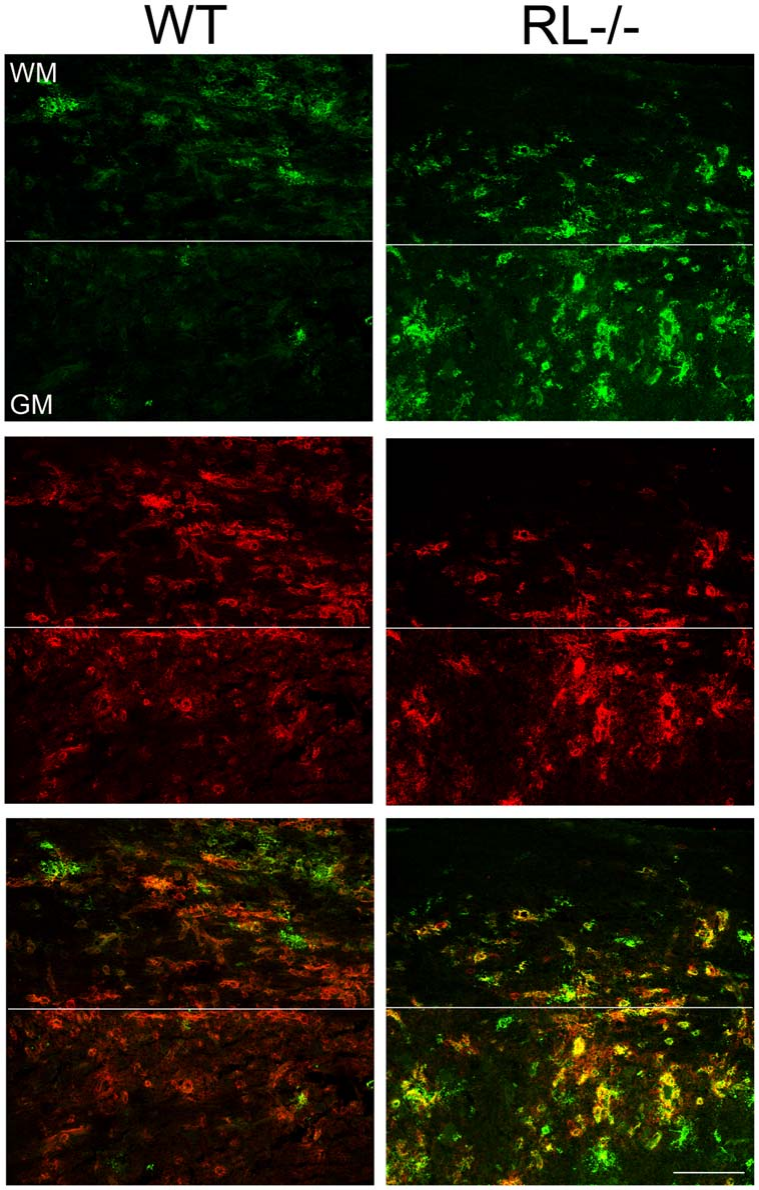
RNase L protects microglia/macrophages from infection in grey matter. Virus infected cells detected using virus nucleocapsid protein specific mAb J.3.3 (Antigen - green) and macrophage/microglia cell marker Iba-1 (Iba-1 – red) in spinal cords from infected wt and RL^−/−^ mice at day 7 p.i. Co-localization of viral antigen and Iba-1 staining cells (Merge - yellow) indicates infected microglia/macrophages are present only in white matter (WM) of wt, but both white matter and grey matter (GM) in RL^−/−^ spinal cords. Scale bar = 100 µm.

The inability to directly link increased axonal damage with enhanced neuronal infection remains puzzling and supports dysregulation of oligodendrocyte function and/or neuroprotective functions of microglia as contributing factors to the severe pathological phenotype and clinical outcome. Increased demyelination in spinal cords has been correlated with the extent of white and grey matter infection in mice infected with heterologous MHV strains [33]. Furthermore increased apoptosis, specifically in spinal cord grey matter of RL^−/−^ mice supported grey matter infection. Increased demyelination as well as apoptosis in grey matter in RL^−/−^ mice thus prompted a more detailed investigation of viral antigen distribution in spinal cords, where the demarcation between grey and white matter is linear as opposed to the more complex organization of brain stem. Neuronal infection was not observed in spinal cords of either RL^−/−^ or wt mice. Furthermore, the number of infected cells with oligodendrocyte morphology in the white matter of RL^−/−^ mice was similar to wt mice at day 7 p.i. (Fig. 8). However, a prominent difference in viral antigen positive cells was noted in spinal cord grey matter. RL^−/−^ mice harboured several foci of infected cells with microglia morphology, whereas viral antigen positive cells were rarely observed in spinal cord grey matter of wt mice (Fig. 8). Furthermore, a large number of infected cells in the grey matter of RL^−/−^ mice were identified as microglia and/or infiltrating monocytes based on co-localization with Iba-1 positive cells (Fig. 9). Foci of Iba-1 positive cells co-expressing viral antigen in the grey matter were only observed in RL^−/−^ but not wt mice, confirming results obtained by immunohistochemistry (Fig. 8). Nevertheless, infected Iba-1 positive cells were dispersed throughout the white matter of wt and RL^−/−^ mice. Similar characterization of the brain stem at day 10 p.i. also identified Iba -1 positive cells as prominent cells harbouring sustained viral infection (data not shown). Co-stains with the astrocyte specific marker GFAP showed no evidence of infected astrocytes in spinal cord grey matter; astrocytes were also only rarely infected in white matter of wt and RL^−/−^ mice (data not shown). These data indicate that RNase L mediated antiviral function acts specifically in microglia and/or monocytes. Moreover the focal nature of infection in grey matter suggests microglia localization is associated with enhanced susceptibility to infection in the absence of RNase L.

To determine whether infection of Iba-1 positive cells was biased to microglia or infiltrating monocytes, both cell populations were purified from spinal cords of infected mice by fluorescence activated cell sorting. Measurement of viral mRNA encoding the nucleocapsid protein revealed that microglia derived from RL^−/−^ mice harbored 3.0-fold higher levels of viral RNA at day 7 p.i., while the relative levels in monocytes was only increased 1.6-fold relative to infected wt mice (Table 2). However, viral mRNA levels decreased in both cell types reaching comparable levels by day 10 p.i. While RNA levels for TNF were elevated in RNase L deficient microglia and monocytes by 2-fold and 1.4 fold, respectively, iNOS levels remained similar relative to wt derived cells (data not shown). IFNγ relative to GAPDH mRNA levels in spinal cord were also modestly increased from 4.6±0.5 in wt mice to 8.1±0.3 in RL^−/−^ mice at the peak (day 7 p.i.) and declined to 1.4±0.4 and 1.2±0.1, respectively, by day 10 p.i., confirming a modest contribution of proinflammatory cytokines to pathogenesis. Moderately increased viral RNA levels in microglia are thus consistent with foci of microglia infection in spinal cord grey matter not present in wt mice.

**Table 2:** Levels of viral nucleocapsid protein encoding mRNA in cell populations purified from spinal cords.

Day p.i.	Microglia		Monocytes	
	wt	RL−/−	wt	RL−/−
7	198^a^±25^b^	577±204	79±14	126±45
10	47±24	60±27	27±1	34±1

a relative to GAPDH.

b SD from two sorting experiments with pooled samples from n = 8 per group and time point.

**Figure 10:**
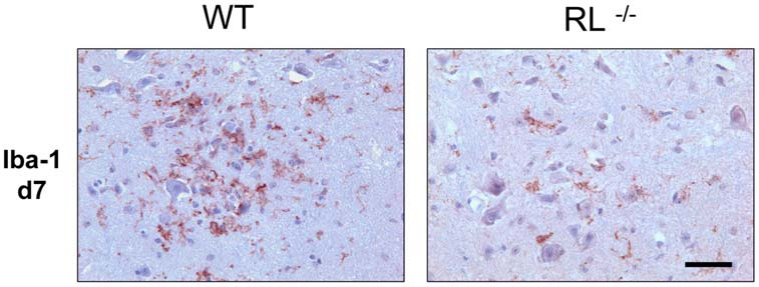
RNase L enhances microglia activation. Microglia activation in spinal cords from infected wt and RL^−/−^ mice at day 7 p.i. Microglia/monocytes detected by immunoperoxidase staining using anti Iba-1antibody.

Overall these data demonstrate that accelerated and enhanced demyelination in the absence of RNase L coincided with significant neuronal damage in the absence of expanded neuronal infection. Furthermore, the limited extent of increased focal glial infection appeared insufficient to mediate the pathological effect. To assess potential differences in the overall activation state of microglia, the distribution and morphology of Iba-1 positive cells was assessed in spinal cord grey matter. Microglia in wt mice were activated as demonstrated by their intense staining and ramified phenotype and localized in close proximity to neuronal cell bodies (Fig. 10). By contrast, in infected RL^−/−^ mice, fewer microglia were activated based on their Iba-1 staining and morphology and did not show preferential association with neurons. Whether the absence of RNase L itself or infection of microglia mitigates a neuroprotective function of microglia remains to be explored. The notion that inflammatory insults disrupt neuroprotective functions by microglia is based on microglia mediated neuroprotection in models of injury and LPS preconditioning [34]–[36] and loss of protection following viral infection [37].

## Discussion

IFN-α/β dependent innate immunity is essential to contain viral spread during most viral infections prior to control by adaptive responses. Nevertheless, the *in vivo* anti-viral effector mechanisms contributing to innate viral control remain poorly understood. The best characterized pathways are mediated by activation of PKR and RNase L [3]–[5]. However, in contrast to the conclusive effects of PKR and RNase L deficiency on viral replication *in vitro*, *in vivo* studies demonstrate more subtle anti-viral effects [8],[9],[11]. During WNV infection, RNase L deficiency is manifested in profoundly altered morbidity, despite similar viral control after footpad inoculation [11]. IFN-α/β-mediated anti-viral responses are also critical for controlling spread of neurotropic coronavirus within glial cell populations and preventing infection of neurons [12]. Based on its activation by numerous RNA viruses, RNase L was investigated as a prototypical anti-viral effector molecule in controlling MHV-JHM virus replication in the CNS.

RNase L deficiency was not sufficient to duplicate uncontrolled replication and the rapid uniform lethality observed in IFNAR^−/−^ mice [12]. Nevertheless, a critical contribution to virus susceptibility was demonstrated by a significant increase in both clinical disease and mortality. Mortality was delayed by 3–4 days in RL^−/−^ relative to IFNAR^−/−^ mice, but could not be attributed to uncontrolled viral replication or spread to neurons. In fact, overall virus replication in brains and spinal cords of RL^−/−^ mice was only modestly increased and declined with kinetics similar to wt mice. These latter findings were reminiscent of transiently increased viral replication in the CNS of RL^−/−^ mice following intracerebral WNV inoculation [11].

In addition to direct anti-viral activities, activated RNase L degrades viral and host RNA [38],[39]. The cleavage products may in turn activate RIG-I/Mda-5 cytoplasmic pattern recognition receptors, propagating the expression of IFN-α/β and ISGs [7]. While this function of RNase L is indeed evident by reduced IFN-β production in EMCV infected RL^−/−^ mice [7], MHV-JHM infection of the CNS presented no evidence for this pathway. The negligible affects of RNase L deficiency on overall viral replication and IFN-α/β mediated responses suggest that MHV-JHM infection in the CNS does not, or only sparsely, activates OAS/RNase L activity *in vivo*. This finding is reminiscent of *in vitro* studies with the heterologous MHV-A59 strain [26]. Nevertheless, the preferential susceptibility of RNase L deficient microglia/monocytes to MHV-JHM infection demonstrates that viral RNA does activate cellular RNA sensors, albeit in a cell type specific manner. This is supported by Mda-5 triggered activation of the IFN-α/β pathway in microglia and macrophages infected with MHV-A59 *in vitro* [17]. Whether the apparent inability of gliotropic MHV-JHM to activate RNase L in other cell types *in vivo* resides in distinct basal OAS/RNase L expression levels, activation of distinct OAS enzymes, or other RNA sensing receptors such as Mda-5 has not been elucidated.

Numerous functions of RNase L, not directly associated with anti-viral activity, may contribute to the increased susceptibility of RL^−/−^ mice to MHV-JHM infection. RNase L plays a role in translational inhibition, regulation of mRNA stability, apoptosis, proliferation and tumor suppression [25],[40]. For example, RNase L contributes to IFN-α/β mediated apoptosis, as well as homeostasis of thymocytes and splenocytes in young naïve mice [8]. RNase L deficiency also delays tissue graft rejection [22] implicating a defect in T cell function or trafficking. Nevertheless, both histochemical and flow cytometric analysis revealed a similar extent and composition of inflammatory cells in the CNS of infected RL^−/−^ and wt mice. The lack of RNase L mediated alterations in T cells was supported by similar peripheral expansion of virus-specific CD8 T cells and kinetics of viral control. Increased morbidity could thus not readily be attributed to altered inflammation or pro-inflammatory signals at the tissue level.

Neuronal infection by gliotropic MHV-JHM is sparse and only evident early p.i. in wt mice. No evidence for enhanced neuronal infection in RL^−/−^ mice thus suggested that the IFN-α/β mediated anti-viral mechanisms in neurons are RNase L independent. This contrasts with a protective role for RNase L in inhibiting HSV-1 replication in neurons of IFN-β treated trigeminal ganglia [41], and supports virus specific susceptibilities to innate anti-viral immunity. Distinguishing features in MHV-JHM infected RL^−/−^ mice are the sustained areas of microglia infection in brain stem and focal areas of infected microglia within the spinal cord grey matter. Infected cells in spinal cord grey matter are also evident in SCID [42] and IFNAR^−/−^ mice (unpublished observation) following infection with the gliotropic MHV-JHM. In RL^−/−^ mice the prominent infected cells in brain stem and spinal cord grey matter were identified as Iba-1 positive microglia or infiltrating monocytes, which cannot readily be distinguished by immunohistochemistry. Nevertheless the morphology of infected cells in the grey matter was consistent with activated microglia, rather than the monocyte/macrophages with dense cytoplasm found prominently in demyelinating lesions. Furthermore, preferential microglia infection was supported by a relatively greater increase of viral RNA in microglia relative to monocytes, when comparing RL^−/−^ versus wt mice. The prominent location of Iba-1 positive cells in grey matter of RL^−/−^ mice could not be attributed to the absence of activated microglia in grey matter of wt mice. In fact, microglia in wt mice exhibited a more ramified phenotype surrounding neurons compared to microglia in RL^−/−^ infected mice. It remains unclear whether abrogation of the proximal localization of microglia to neurons in RL^−/−^ spinal cords reflects an unknown function of an RNase L enhanced neuroprotective effect, or altered function due to infection.

Surprisingly, both prolonged brain stem infection and enhanced spinal cord grey matter infection was associated with more severe demyelination and axonal damage. Overall, the CNS pathology characteristic of MHV-JHM infection was accelerated by 3–4 days in RL^−/−^ relative to wt mice. Although infection of microglia correlated with a subsequent increase in apoptotic cells, apoptotic cells surrounding neurons were only evident in spinal cord, not in brain stem. The inability to detect increased numbers of infected or apoptotic cells in spinal cord white matter is consistent with both an apparent lack of RNase L activation in oligodendrocytes during MHV-JHM infection and paucity of apoptotic oligodendrocytes in wt mice [28].

Whether apoptotic cells originated from infected myeloid cells themselves or from lymphocytes migrating to the infected areas and exerting effector function is unclear. Preliminary analysis showed no co-localization of activated caspase 3 and Iba-1 (data not shown). Overall, the neurologic disability and morbidity of the infected RL^−/−^ mice appears to result from axonal or neural degeneration, independent of neuronal infection in both brain stem and spinal cord. The enhanced demyelination phenotype is thus distinct from demyelination attributed to enhanced white matter infection by recombinant neuronotropic MHV variants [33]. RNase L deficiency and infection of microglia may contribute to this pathology in several ways. Infection in the absence of RNase L may impair neuroprotective effects exerted by microglia under inflammatory conditions [34]–[36]. Alternatively, enhanced infection of microglia may increase proinflammatory responses resulting not only in enhanced recruitment of T cells and monocytes, but also increased local production of neurotoxic factors such as TNF, nitric oxide, oxidative radicals, and matrix metalloproteases. However, only modest increases in IFNγ mRNA in spinal cord, as well as minor differences in TNF and iNOS mRNA in microglia/monocytes suggest these effects may only be apparent at a focal level. Increased localized T cell effector function, manifested in release of perforin and granzymes, has also been shown to contribute to axonal injury without affecting demyelination [30]. Lastly, RNase L deficient and/or infected microglia may perturb normal microglia functions in maintaining neuronal health as indicated by disruption of neuronal autophagy by SIV infected microglia [37]. It thus remains to be determined to what extent accelerated and more severe pathology is due to disruption of the neuroprotective functions of microglia and/or from overt activation due to infected cells [43]–[47]. Independent of a pathogenic effect, it is interesting to note that the absence of RNase L also enhanced macrophage susceptibility to WNV infection [11]. Whether this reflects differential activation of OAS enzymes or participation of other pattern recognition receptors such as Mda-5 in susceptible cell types *in vivo* remains to be elucidated.

In summary, these data highlight a novel role of RNase L in protection from virus induced demyelination. Although RNase L did not play an overt anti-viral role as measured by viral replication, it did provide specific protection from focal infection of microglia/macrophages in the CNS. RNase L was not crucial in protecting neurons from infection and played no obvious role in protecting oligodendrocytes or astrocytes. Furthermore, RNase L deficiency did not alter proinflammatory responses, diminish IFN-α/β mediated signals, or dampen adaptive immune mediators. The results rather uncover how subtle local alterations in viral tropism may affect the balance between neuroprotection and neurotoxicity mediated by microglia/macrophages.

## Materials and Methods

### Animals, viruses, and clinical scores

C57BL/6 mice were purchased from the National Cancer Institute (NCI, Fredrick, MD). C57BL/6-RL^−/−^ mice were bred and housed under pathogen-free conditions in the Biological Resources Unit of the Cleveland Clinic. All procedures were performed in compliance with protocols approved by the Institutional Animal Care and Use Committee. Mice were infected at 6 weeks of age by intracranial injection with 250 PFU of the gliotropic MHV-JHM variant V2.2-1 [48] in 30 µL endotoxin-free Dulbecco's phosphate buffered saline (DPBS). The severity of clinical disease was graded as previously described [48]: 1 = ruffled fur, 2 = slow righting reflex, 3 = loss of righting reflex, 4 = moribund. Following intraperitoneal administration of ketamin/xylaxine (100 mg/kg/10 mg/kg), mice were perfused intracardially with 10 mL DPBS. Brains, spinal cords, spleens, cervical lymph nodes (CLN) and livers were collected and processed as described below.

### Virus titer and cell isolation

Brains were bisected sagittally. One half-brain and whole spinal cord from each mouse were homogenized in ice cold Tenbroeck glass grinders in 4 mL or 2 mL of DPBS, respectively. Homogenates were clarified by centrifugation for 7 min at 400×g. Supernatants were stored at −80°C. Virus in supernatants was measured by plaque assay on monolayers of delayed brain tumor (DBT) astrocytoma cells as previously described [48]. CNS cells from homogenate pellets were resuspended in RPMI containing 25 mM HEPES, adjusted to 30% Percoll (Pharmacia, Upsalla, Sweden), underlayed with 1 mL 70% Percoll, centrifuged at 800×g for 30 minutes and collected from the 30%/70% Percoll interface as previous described [23]. Purified CNS cells were washed and resuspended in RPMI.

### Flow cytometry and Fluorescence Activated Cell Sorting

Cell populations isolated from the brain and spinal cord were phenotyped using four-color flow cytometry. Prior to staining, cells were incubated with 1% mouse serum and 1% rat anti-mouse FcγIII/IIR monoclonal antibody (mAb) in fluorescent antibody cell sorting (FACS) buffer (0.5% bovine serum albumin in DPBS) for 20 minutes at 4°C to block non-specific binding. Cell types were identified using fluorescein isothiocyanate-, phycoerythrin-, peridinin-chlorophyll-protein complex- or allophycocyanin-conjugated anti-mouse mAb: Ly-6 g (1A8), CD4 (GK1.5), CD8 (53.67) (all from BD Biosciences, San Diego, CA) and F4/80 (CI:A3-1; Serotec, Raleigh, NC). Virus specific CD8 T cells were identified using H-2D^b^/S510 MHC class I tetramers as described previously [23]. Cells were incubated with antibodies for 30 minutes on ice, washed twice with FACS buffer and fixed with 2% paraformaldehyde for 10 minutes on ice. At least 100,000 events were acquired on a FACSCalibur flow cytometer (BD Biosciences, San Jose, CA) for subsequent data analysis using Flow-Jo 7 Software (Tree Star, Inc. Ashland, OR).

For fluorescence activated cell sorting of microglia and monocyte populations, spinal cords from eight mice per group were finely minced using a razor blade and dissociated in a 0.25% trypsin solution as described [27],[49] at 37°C for 30 minutes with periodic tituration. Trypsin was quenched by addition of RPMI supplemented with 25 mM HEPES and 20% new born calf serum. The dissociated cells were washed in RPMI containing 25 mM HEPES, 1% FCS, then isolated from the interphase of a 30%/70% percoll gradient as described above. Cells were incubated with 1% mouse serum and CD16/32 prior to staining with allophycocyanin-conjugated mAb specific for CD45 (30-F11), peridinin-chlorophyll protein-conjugated CD11b (M1/70) (BD Biosciences, San Diego, CA) and phycoerythrin-conjugated mAb specific for F4/80 (CI:A3-1; Serotec, Raleigh, NC). Monocyte/macrophages and microglia were purified on a FACSAria cell sorter (BD Biosciences, San Diego, CA) based on their respective CD45^hi^CD11b^+^F4/80^+^ and CD45^lo^ CD11b^+^F4/80^+^ phenotypes.

### Histopathological analysis and fluorescence immunocytochemistry

One half-brain and whole spinal cords were fixed with formalin and embedded in paraffin. Sections were stained with either hematoxylin and eosin or luxol fast blue as described [50]. Distribution of viral antigen was determined by immunoperoxidase staining (Vectastain-ABC kit; Vector Laboratory, Burlingame, CA) using mAb J.3.3, specific for the carboxyl terminus of the viral nucleocapsid protein as primary antibody, biotinylated horse anti-mouse as secondary antibody, streptavidin-conjugated horse radish peroxidise and 3,3′-diaminobenzidine substrate (Vector Laboratory) [50]. Similarly, immunoperoxidase staining for neurofilament used mouse anti-phosphorylated and anti-non-phosphorylated neurofilament mAb (SMI-31 and SMI-32, Covance, Princeton, NJ) and immunoperoxidase staining for microglia/macrophages used anti-ionized calcium-binding adapter molecule-1 antibody (Iba-1, Abcam, Cambridge, MA). Apoptotic cells were detected by rabbit anti-activated caspase-3 Ab (Asp175, Cell Signalling Technology, Beverly, MA). Sections were scored for inflammation, viral antigen, apoptotic cells, axonal damage and demyelination in a blinded fashion. Representative fields-of-view were identified based on average scores of all sections in each experimental group. For calculation of the percentage area of demyelination, sections were digitized and analysed as previously described [27].

To identify infected cell types, spinal cords were embedded in TissueTek O.C.T. compound (Andwin Scientific, Tryon, NC), flash frozen in liquid nitrogen and stored at −80°C. Blocks were warmed to −20°C prior to cutting 10 µm sections by cryostat at this temperature. Following fixation with 4% paraformaldehyde for 30 minutes at room temperature, non-specific antibody binding was blocked using 1% bovine serum albumin, 5% normal goat serum. Infected cells were identified using anti-MHV-JHM J3.3 mAb, rabbit anti-glial fibrillary acidic protein antibody (GFAP, Abcam, Cambridge, MA) and rabbit anti-ionized calcium-binding adapter molecule-1 antibody (Iba-1, Wako, Richmond, VA) in combination with goat anti-mouse Alexa-fluor 488 and goat anti-rabbit Alexa-fluor 594-conjugated IgG (Invitrogen, Carlsbad, CA) as secondary antibodies, respectively. Sections were mounted with ProLong Gold antifade mounting media containing 4′,6-diamidino-2-phenylindole (Invitrogen, Carlsbad, CA). Imaging of immunofluorescent sections was performed with a Leica SP-5 confocal microscope (Leica Microsystems, Bannockburn, IL). Z-projections of sections were processed by ImageJ software (National Institutes of Health, Bethesda, MD)

### Real-time PCR

One-half brains and whole spinal cords were snap frozen in liquid nitrogen and stored at −80°C. Frozen brain or spinal cord tissue was homogenized in 2 mL and 1 mL Trizol reagent (Invitrogen, Carlsbad, CA), respectively, in RNase-free Tenbroeck glass grinders. RNA was purified according to the manufacturer's protocol (Invitrogen, Carlsbad, CA). Briefly, 0.2 mL chloroform/1 mL trizol (Sigma-Aldrich, St. Louis, MO) was added to homogenate, mixed and centrifuged at 12,000×g for 15 minutes at 4°C. RNA was precipitated from the aqueous phase by addition of isopropyl alcohol and centrifugation at 12,000×g for 10 min at 4°C washed in RNase-free 75% ethanol and resuspended in Ultrapure DNase/RNase-free water (Invitrogen, Carlsbad, CA). Cells isolated by FACS were immediately resuspended in 400 µL of Trizol reagent (Invitrogen, Carlsbad, CA) and treated as above. Isolated total RNA was treated with DNase1, using the DNA-free kit (Applied Biosystems, Foster City, CA) following the manufacturer's protocol. The concentration and purity of RNA was measured by spectrophotometry at 260/280 nm. RNA integrity was confirmed by 1.2% formaldehyde-agarose gel electrophoresis.

Reverse transcription was performed on 2 µg total RNA isolated from brain and spinal cord or all total RNA isolated from FACS sorted cells, primed with 250 ng random hexamers, (Invitrogen, Carlsbad, CA) using MMLV reverse transcriptase (Invitrogen, Carlsbad, CA) for 50 minutes at 37°C. Real-time PCR was performed using SYBR Green 2×Master Mix (Applied Biosystems, Foster City, CA) for the following primer sets: Interferon-induced protein with tetratricopeptide repeats (IFIT)-1: F: 5′-CCTTTACAGCAACCATGGGAGA-3′, R: GCAGCTTCCATGTGAAGTGAC-3′; IFIT-2 (ISG-54): F: 5′-AGAGGAAGAGGTTGCCTGGA-3′, R: 5′-CTCGTTGTACTCATGACTGCTG-3′; OAS2: F: 5′-AAAAATGTCTGCTTCTTGAATTCTGA-3′, R: 5′-TGTGCCTTTGGCAGTGGAT-3′; JHMV-N: F: 5′-TAAGAGTGATTGGCGTCG-3′, R: 5′-ATTGGGTTGAGTAGTTGCAG-3′; iNOS: F: 5′- CCTGGTACGGGCATTGCT-3′, R: 5′-CATGCGGCCTCCTTTGAG-3′; TNF-α: F: 5′- GCCACCACGCTCTTCTGTCT-3′, R: 5′- GGTCTGGGCCATAGAACTGATG-3′ and for endogenous control, GAPDH: F: 5′ -CATGGCCTTCCGTGTTCCTA-3′, R: 5′-ATGCCTGCTTCACCACCTTCT-3′. The reaction conditions were: 95°C for 10 minutes, followed by 40 cycles of: denaturation at 95°C for 10 seconds, elongation at 60°C for 30 seconds and annealing at 72°C for 30 seconds. IFN-β1, IFN-α4 and IFN-γ levels were determined by real time PCR using ABI Gene Expression Assays with 2×Universal Taqman Fast Master Mix (Applied Biosystems, Foster City, CA), using manufacturer default cycling conditions and GAPDH expression as an endogenous control. All real-time reactions were run using an ABI 7500fast real-time cycler and analysed with 7500fast software (Applied Biosystems, Foster City, CA). Data presented are expressed as fold-induction relative to GAPDH based on the following formula: (2^(CT(GAPDH)-CT(TARGET))^)*1000.

### Statistical analysis

Students' t-Test with equal variance was used to compare RL^−/−^ and wt C57BL/6 mice. Significant differences between groups are noted by: *,  = P≤0.05.
